# The Double-Edged Sword Effect of Empathic Concern on Mental Health and Behavioral Outcomes: The Mediating Role of Excessive Adaptation

**DOI:** 10.3390/bs15040463

**Published:** 2025-04-03

**Authors:** Rui Wang, Xuanyu Zhang, Lixin Zhu, Huina Teng, Dengdeng Zhang, Boyu Qiu

**Affiliations:** 1 School of Health Management, Guangzhou Medical University, Guangzhou 510180, China; 2 School of Mental Health, Guangzhou Medical University, Guangzhou 510180, China

**Keywords:** empathic concern, excessive adaptation, mental health, prosocial behaviors, reactive aggression, depression

## Abstract

This study examines the complex effects of empathic concern on mental health and behavioral manifestations and the potential indirect paths through excessive adaptation. A cross-sectional design with 1355 participants was employed. Empathic concern, excessive adaptation, prosocial behaviors, reactive aggression, depression, and positive mental health were assessed using established scales. Structural equation modeling and Bayesian linear regression were applied to analyze the paths. For direct paths, empathic concern positively predicted prosocial behaviors and positive mental health, whereas it was negatively related to depression and reactive aggression. For indirect paths, excessive adaptation was found to mediate the relationship between empathic concern and the outcome variables with the exception of positive mental health. By elucidating the mediating role of excessive adaptation, the results herein not only deepen our understanding of the dual effect of empathic concern on mental health and behavioral manifestations but also offer important insights for the medical and educational fields.

## 1. Introduction

Empathy, a multidimensional construct in human social cognition, encompasses both affective and cognitive dimensions (Decety & Jackson, 2004). The multidimensional model of empathy proposed by Davis (1983a) divides empathy into cognitive and affective pathways; perspective-taking and fantasy belong to cognitive empathy, while empathic concern and personal distress are categorized under affective empathy. As a key component, empathic concern indicates an individual’s tendency for the respondent to experience feelings of warmth, compassion, and concern for others undergoing negative experiences. In contrast to positive emotions, personal distress reflects self-oriented anxiety arising from witnessing the suffering of others. Both are underpinned by distinct psychobiological mechanisms, including neural circuits and psychophysiological activity (Chierchia & Singer, 2017; Decety & Lamm, 2009; O. M. Klimecki et al., 2013, 2014). Furthermore, Cuff et al. (2016) found that concepts related to empathic concern and compassion are often conflated in the literature. Although both are associated with prosocial behaviors (Davis, 1983b; Leiberg et al., 2011), empathic concern elicits emotions similar to those of others through affective contagion, focusing on emotional understanding of others. Conversely, compassion fosters concern and empathy closer to those of others, highlighting the translation of these feelings into a lasting commitment to action through cognitive regulation (Gleichgerrcht & Decety, 2013).

Empathic concern is of great importance in human socialization and sustaining interactions in groups and societies; it enables us to perceive the emotions and perspectives of others (Batson, 2023). The empathy–altruism hypothesis (Batson et al., 1991; Persson & Kajonius, 2016) and the dual-process model (Greene et al., 2004; Patil & Silani, 2014) propose that the affective dimensions of empathy, such as empathic concern, play a vital role in moral judgement (Decety & Cowell, 2014). In neurobiological research, Coutinho et al. (2014) argued that empathic concern involves complex interactions between multiple brain regions, the autonomic nervous system, and the neuroendocrine system. These brain regions facilitate mentalizing (Astington & Hughes, 2013) and play a crucial role in integrating cognitive and emotional abilities (Decety & Svetlova, 2012). Similar results can be found in cross-cultural research (Atkins et al., 2016; Zheng et al., 2024) and laboratory studies (Twenge et al., 2007; Wölfer et al., 2012). Based on its function on moral judgement and mentalizing, the role of empathic concern on mental health and behavioral manifestations has been mentioned in a series of studies.

Previous studies have explored the positive effects of empathic concern. For instance, Qiu et al. (2024) indicated that empathic concern is related to increased prosocial behaviors in meta-analysis. The empathy–altruism hypothesis further confirms empathic concern as a source for more prosocial behaviors and motivational beliefs (Persson & Kajonius, 2016). There is also evidence that empathic concern can promote individual mental health by influencing emotional regulation (Schipper & Petermann, 2013; Zaki, 2020). In a systematic review and meta-analysis study, He et al. (2023) also pointed out that empathic concern was positively related to mental health and subjective well-being. In a word, the bright side of empathic concern has a solid research foundation.

As a complex ability (De Waal & Preston, 2017), the dark side of empathic concern was also studied. The vulnerability model (Tone & Tully, 2014) posits that affective empathy can contribute to the emergence of internalizing problems, including anxiety (McGrath et al., 2012) and depression (Cicchetti & Toth, 2009). There is also evidence that empathic concern can lead to depression as a result of overbearing the emotions of others (Figley, 1995) or the breakdown of moral idealization (Decety & Cowell, 2014). Gambin and Sharp (2018) discovered that empathic concern can intensify depressive symptoms by triggering excessive feelings of responsibility and guilt. Ding et al. (2024) confirmed a positive correlation between empathic concern and depression through a three-wave random intercept cross-lagged panel analysis. Therefore, it can be argued that empathic concern is also detrimental (Shamay-Tsoory, 2011; Stefanello, 2022).

However, few studies have explored the external behavioral manifestations of the dark side. On one hand, empathic concern might become detrimental by causing increased motivation to protect oneself (Song et al., 2018), which in turn contributes to emotional over-arousal (Dodge & Coie, 1987). On the other hand, individuals experience empathic concern in a way that is personally distressing and accompanied by excessive negative emotions (Eisenberg et al., 2010). Both emotional over-arousal and negative valence can lead to a greater possibility of engaging in reactive aggression (Anderson & Bushman, 2002; Tampke et al., 2020). Moreover, empathic concern can lead to internal problems, thereby increasing reactive aggression (Lovett & Sheffield, 2007). For example, Gill and Calkins (2003) observed that children with higher levels of aggression responded faster to others’ distress and exhibited greater empathic concern (Gill & Calkins, 2003). Therefore, we hypothesize that empathic concern can lead to negative externalizing behavioral manifestations.

Empathic concern can lead individuals to excessively adapt to the needs of others at the cost of their own well-being, potentially leading to burnout and emotional distress as a result of self-sacrifice (Hong & Wang, 2024; Y.-Y. Zhang et al., 2018). Excessive adaptation, defined as a state of imbalance arising from excessive accommodation to external demands (Abe et al., 2020), has attracted widespread attention in multiple disciplines. Excessive adaptation is a concept that has been discussed more in studies focusing on East Asian populations, where collectivist norms may amplify tendencies to prioritize the demands of others (Markus & Kitayama, 1991).

According to attachment theory, the internal working model of insecurely attached individuals makes them hypersensitive to others’ evaluations (Ren, 2022) and maintains relational security through constant monitoring of others’ needs and ritualized giving behaviors (Mashiko, 2009). This model forces individuals to be overly concerned with the needs of others, triggering self-need repression and emotional exhaustion, which ultimately leads to depression and obsessive–compulsive symptoms through chronic stress (Kosaka, 2008). Therefore, individuals with high levels of excessive adaptation are more prone to depression and obsessive–compulsive disorders compared to the general population. Studies have also pointed to a negative correlation between excessive adaptation and subjective well-being (Asai, 2014; Tsuda, 2021). Therefore, this study posits that excessive adaptation can mediate the relationship between empathic concern and both positive and negative outcomes.

**Figure 1:**
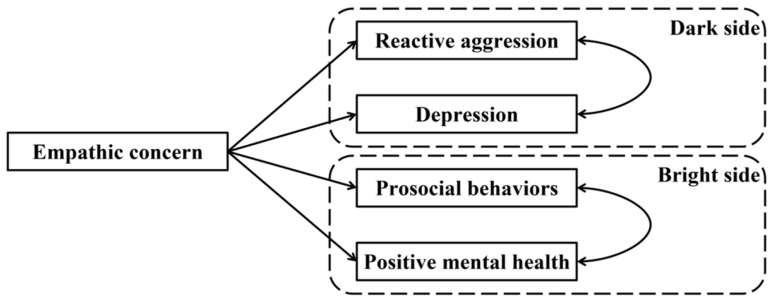
Full proposed model about two aspects of empathic concern.

**Figure 2:**
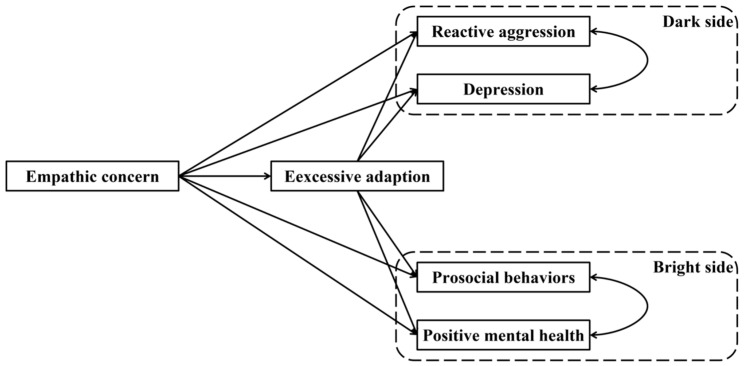
Full proposed model for the mediating role of excessive adaptation.

Based on existing research, the diverse outcomes of empathic concern underscore the complexity and diverse nature of this psychological phenomenon, which can manifest and be internalized in various ways (Levy et al., 2019). Investigating and effectively harnessing empathic concern, a double-edged sword, is of significant importance for human socialization. Our study specifically highlights the mediating role of excessive adaptation in this context. To address these gaps and further explore empathic concern, we proposed the following:

(1) Empathic concern is a double-edged sword. Empathic concern has both positive and negative dimensions, impacting both internal mental health and external behavioral manifestations. While it can foster prosocial behaviors and enhance positive mental health, it can also lead to reactive aggression and depression (Figure 1).

(2) Excessive adaptation has a mediating role. Excessive adaptation mediates the relationship between empathic concern and its diverse outcomes (Figure 2). Specifically, individuals with high levels of empathic concern are more likely to engage in excessive adaptation, which can intensify negative outcomes (i.e., reactive aggression and depression) while potentially diminishing positive outcomes (i.e., prosocial behaviors and positive mental health).

## 2. Methods

### 2.1. Study Design

This study assessed six key variables: empathic concern, prosocial behaviors, reactive aggression, excessive adaptation, depression, and positive mental health. All six variables were assessed using validated scales.

### 2.2. Setting

Data were collected through structured self-report questionnaires. Participants first submitted informed consent forms and then completed the questionnaires. The settings of this study received approval from the Research Ethics Committee of the institution with which the first author is affiliated.

### 2.3. Participants

Participants were recruited from local universities. After our screening process through lie detector questions, the final sample consisted of 1355 valid questionnaires, including 928 male students (68.49%) and 427 female students (31.51%), with a mean age of 21.28 years (*SD* = 1.78, range: 18–28 years). Informed consent forms, detailing the study’s purpose, the voluntary basis of participation, and the commitment to data privacy, were received from participants.

### 2.4. Measures

#### 2.4.1. Empathic Concern

Empathic concern was measured using the empathic concern subdimension from the Chinese version (F. F. Zhang et al., 2010) of the interpersonal reactivity index (Davis, 1983a). The empathic concern subdimension includes 6 items, for example, “I often feel tender-hearted and caring towards those who are less fortunate than me”, “When I see someone being taken advantage of, I feel an urge to protect them”, and “I consider myself to be quite a soft-hearted person”. Respondents were asked to rate items on a five-point scale (1 = not at all, 5 = extremely). The total score was calculated, with higher scores indicating greater empathetic abilities. In this study, the Cronbach’s alpha of the empathic concern items was 0.691.

#### 2.4.2. Prosocial Behaviors

Prosocial behaviors were measured by the Chinese version (Kou & Wang, 2003) of the prosocial tendencies measure (Carlo & Randall, 2002). The scale comprises 26 items, and 6 dimensions—emotional (e.g., “When I can comfort someone who is feeling down, I feel very good”), compliant (e.g., “When others ask for my help, I rarely refuse”), anonymous (e.g., “I prefer to make donations anonymously”), public (e.g., “When people are around, I will go all out to help others”), urgent (e.g., “I am inclined to help those who are truly in trouble and urgently need assistance”), and altruistic (e.g., “I donate money and goods not for any benefit in return”)—were included in the scale. Respondents were required to rate the extent to which the item descriptions matched their own situations over the past three months, using a five-point scale (1 = completely disagree, 5 = completely agree). The higher the total score, the greater the degree of the participant’s prosocial behaviors. The Cronbach’s alpha for the scale in this study was 0.948.

#### 2.4.3. Reactive Aggression

Reactive aggression was measured using the reactive aggression subdimension from the Chinese version (W. L. Zhang et al., 2014) of the reactive–proactive aggression questionnaire (Raine et al., 2006). The reactive aggression subdimension includes 10 items, for example, “When someone annoys me, I yell at them”, “When I encounter obstacles or setbacks, I get very angry”, and “I hurt others in order to win the competition”. Respondents were asked to rate items on a three-point scale (1 = never, 2 = sometimes, 3 = often), and the scores were summed, with higher scores indicating stronger aggression behaviors. The Cronbach’s alpha of the reactive–proactive aggression questionnaire was 0.940 in this study.

#### 2.4.4. Excessive Adaptation

Excessive adaptation was measured using the excessive adaptation subdimension from the Chinese version (X. Wang, 2021) of the over-adaptation scale (Ishizu & Ambo, 2008). Excessive adaptation is divided into 3 subdimensions: consideration for others (8 items, e.g., “thinking about what others expect me to do”), striving to meet expectations (7 items, e.g., “considering what actions will lead to praise from others”), and seeking high evaluation (5 items, e.g., “desiring recognition from others”). Respondents were asked to rate items on a five-point scale (1 = completely disagree, 5 = completely agree), with higher total scores indicating a higher degree of over-adaptation. The Cronbach’s alpha of the excessive adaptation items was 0.930 in this study.

#### 2.4.5. Depression

Depression was measured using the depression subdimension from the Chinese version (Gong et al., 2010) of the depression and anxiety stress scales (Lovibond & Lovibond, 1995). This scale is utilized to assess the emotional conditions of college students. The depression subscale includes 7 items (e.g., “I feel that I have little to look forward to in the future”, “I feel as if I can no longer experience any happiness or comfort”, and “I find it difficult to initiate work”). Respondents were asked to rate items on a four-point scale (0 = not at all, 3 = most of the time), with higher scores signifying greater negative emotional experiences of depression. In this study, the Cronbach’s alpha of the depression items was 0.905.

#### 2.4.6. Positive Mental Health

The positive mental health scale (Lukat et al., 2016) was used to assess the positive aspects of health and life experiences. The scale comprises 9 items, for example, “I often feel carefree and in good spirits”, “I am in good physical and emotional health”, and “I have very well satisfied needs”. Respondents were asked to rate items on a four-point scale (0 = not at all, 3 = always). The scores were summed, with a higher score indicating a higher level of positive psychological health. The Cronbach’s alpha for the scale in this study was 0.903.

### 2.5. Bias

All variables in this study were measured using self-reported questionnaires and may have common method bias; therefore, the data were tested for common method bias using the Harman one-way test prior to analysis (Podsakoff et al., 2003). All six variables were included in an exploratory factor analysis, and the first factor accounted for 37.11% of the total variance, which is lower than the critical value of 40%. Therefore, it was concluded that there was no significant common method bias in the sample data of this study.

### 2.6. Statistical Analysis

The mediating effects were estimated using the SEM module from JASP software 0.19.3.0 (Rogers, 2024). Gender was included as a covariate in the analysis of mediating effects. Model fit was assessed using a variety of fit indices, including the ratio of chi-square over degrees of freedom (χ^2^/df), comparative fit index (CFI), the Tucker–Lewis index (TLI), the root mean square error of approximation (RMSEA), and the standardized root mean square residual (SRMR). The previous literature (Hoyle, 2012) indicates that the model fit is good when χ^2^/df ≤ 5; CFI ≥ 0.95, TLI ≥ 0.95, RMSEA ≤ 0.08, and SRMR ≤ 0.08. The procedure computes an estimation of the indirect effect with a 95% confidence interval (CI). The indirect effect is considered significant when zero is excluded from the confidence interval. Bayesian linear regression analysis was also used to quantify the evidence in favor of the null hypothesis, providing a more comprehensive understanding of the relationships.

## 3. Results

### 3.1. Descriptive Statistics

Means, standard deviations, and correlations across all variables are presented in Table 1. Correlation analysis indicated that empathic concern was positively related to excessive adaptation, prosocial behaviors, and positive mental health (*r*1 = 0.122, *r*2 = 0.331, *r*3 = 0.137; *p*s < 0.001). Empathic concern was negatively related to reactive aggression (*r* = −0.372; *p* < 0.001) and depression (*r* = −0.248; *p* < 0.001). A correlation matrix is also presented in Table 1; the variables included in this study were significantly related to each other (*p*s < 0.001).

### 3.2. Diverse Effects of Empathic Concern

**Figure 3:**
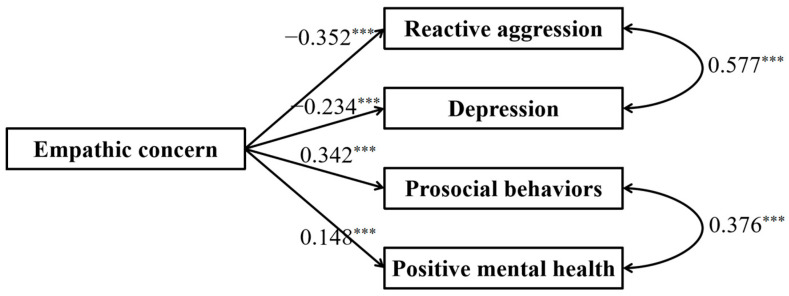
Results of the full proposed model of two aspects of empathic concern. Note: significant standardized paths are displayed by the solid lines. *** *p* < 0.001.

Figure 1 displays the full proposed model for the dark and bright side of empathic concern, including paths from empathic concern to prosocial behaviors, positive mental health, reactive aggression, and depression. The standardized path coefficients are presented in Figure 3. The results showed that empathic concern negatively predicted reactive aggression and depression and positively predicted prosocial behaviors and positive mental health.

### 3.3. The Mediating Effect of Excessive Adaptation

**Figure 4:**
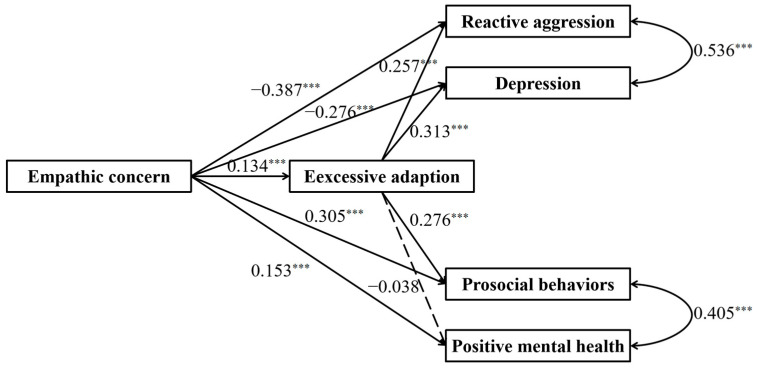
Results of the full proposed model of the mediating role of excessive adaptation. Note: Significant standardized paths are displayed by solid lines. Paths that did not reach the significance level are indicated by dashed lines. *** *p* < 0.001.

**Figure 5:**
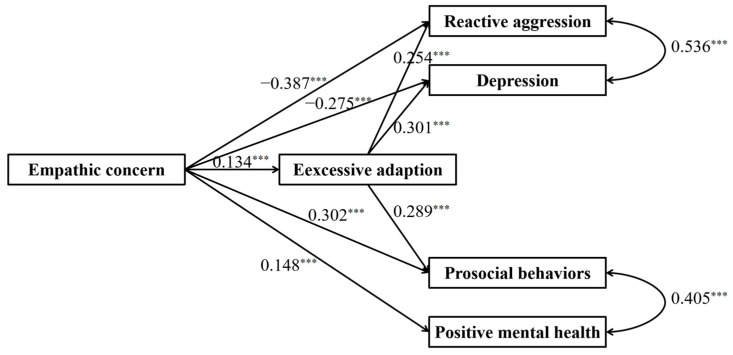
Results of the alternative model of the mediating role of excessive adaptation. Note: significant standardized paths are displayed by the solid line. *** *p* < 0.001.

The full proposed model was constructed, including (a) direct paths from empathic concern to prosocial behaviors, positive mental health, reactive aggression, and depression, (b) indirect path: empathic concern → excessive adaptation → prosocial behaviors, (c) indirect path: empathic concern → excessive adaptation → positive mental health, (d) indirect path: empathic concern → excessive adaptation → reactive aggression, (e) indirect path: empathic concern → excessive adaptation → depression (Figure 2). The path from excessive adaptation to positive mental health did not reach a significant level in the full proposed model (see Figure 4 for standardized path coefficients). Therefore, we removed the path between excessive adaptation and positive mental health to construct an alternative model. The alternative model fitted the data well, χ^2^(df = 1) = 1.913, *p* = 0.167; CFI = 1.000, TLI = 0.991, SRMR = 0.008, and RMSEA = 0.026 (see Figure 5 for standardized path coefficients) and was more concise than the full model. Therefore, the alternative model was selected as the final model. The indirect effects for the full model and their 95% confidence intervals are reported in Table 2. Results showed that the mediation effect of excessive adaptation on paths from empathic concern to prosocial behaviors, reactive aggression, and depression reached a significant level (indirect effects = 0.037, 0.035, 0.042, respectively; *p*s < 0.001), while the indirect effect did not reach a significant level in the path from empathic concern to positive mental health.

### 3.4. Bayesian Linear Regression

Since the path from excessive adaptation to positive mental health did not reach a significant level, the null hypothesis significance testing did not provide sufficient inference for the null hypothesis. To further test the effect of excessive adaptation on positive mental health, we conducted Bayesian linear regression (Wetzels et al., 2011) as a supplementary analysis.

Based on the previous categorization of the magnitude of Bayes factors (Jarosz & Wiley, 2014; Jeffreys, 1961), an inclusion Bayes factor (*BF*incl) lower than 0.33 indicates that the likelihood of *H*0 occurring is three times that of *H*1 in the current data, which provides evidence for the null hypothesis. We conducted Bayesian linear regression on positive mental health, with empathic concern and excessive adaptation as independent variables. The *BF*incl for excessive adaptation was 0.329 in the current study, indicating that excessive adaptation was not related to positive mental health in our sample.

## 4. Discussion

This study investigates the dual aspects of empathic concern, and the results partially support Hypothesis 1. Consistent with previous findings (Fu et al., 2022; He et al., 2023), empathic concern positively predicts prosocial behaviors and mental health. Driven by empathic concern, individuals provide more support to others and are more sensitive to their needs in social interactions (Crocker et al., 2010). Emotional regulation based on empathic concern can enhance positive emotions experienced by individuals and activate the sympathetic nervous system (Guo et al., 2023; Jackson et al., 2000), thereby improving mental health (Berking & Whitley, 2014). However, the results showed that empathic concern is negatively correlated with depression and reactive aggression, which is contrary to previous studies (Lovett & Sheffield, 2007; M. Zhang et al., 2021).

The dark side of empathic concern is not supported in our sample. According to the psychological numbing hypothesis, repeated exposure the stimuli with a high emotional intensity leads to psychological desensitization (Pluta et al., 2023). Consequently, sustained investment in empathic concern may result in empathic concern numbness, thereby reducing its negative effects. Additionally, this study is based on college students, which differs from previous research that is primarily based on children and adolescents (Euler et al., 2017; Strayer & Roberts, 2004). For example, Moreno et al. (2024) showed that personal distress significantly positively predicted aggressive behaviors in convicted adults, mediated by self-compassion. This contrasts with our findings, possibly because college students generally have more developed emotion regulation strategies compared to convicted adults, enabling them to channel empathic concern into problem-solving and prosocial behaviors rather than aggression or self-defense. The relationship between empathic concern and reactive aggression is also moderated by age (Ritchie et al., 2022). As individuals grow older, reactive aggression and depression may be influenced by a more complex array of factors, including situational (Huesmann, 2018) and cognition-related (Allen et al., 2018). Therefore, empathic concern may not necessarily lead to negative outcomes.

It was worth noting that the dual nature of empathic concern is not a static opposition. Positive appraisals of the bright side may mitigate negative impacts through emotion regulation strategies. For instance, cognitive reappraisal preserves the prosocial effects of empathic concern, whereas emotional suppression reduces its efficacy and exacerbates negative social attitudes (Lebowitz & Dovidio, 2015). Such cognitive modulation may create a virtuous cycle. Valuing empathic concern facilitates adaptive emotion regulation, thereby reducing the risk of anxiety and depression. Conversely, excessive focus on the dark side may undermine the positive effects of empathic concern. Individuals who attribute the emotional burden caused by empathic concern to personal vulnerability rather than external circumstances may trigger self-criticism and social withdrawal, ultimately impairing relational functioning. This suggests that irrational interpretations of negative outcomes may suppress the moral motivation inherent to empathic concern unless guided by a rational cognitive framework.

This study also introduces excessive adaptation as a potential mediating variable. The results show that excessive adaptation has a suppressive effect (Kenny et al., 2003; MacKinnon, 2012; Wen & Ye, 2014) in the paths from empathic concern to reactive aggression and depression. Specifically, the direct effects of empathic concern on reactive aggression and depression are negative, whereas the indirect paths through excessive adaptation to depression and reactive aggression were both positive. This suggests that excessive adaptation, to some extent, restores the direct relationship between empathic concern and reactive aggression or depression. The findings suggest that continuous empathic concern may lead to the development of excessive adaptation, which could reduce the positive effects of empathic concern and paradoxically increase the risk of experiencing depression and behaving reactive aggression.

It is worth noting that excessive adaptation and empathic distress are distinct constructs, although both stem from heightened interpersonal sensitivity. Excessive adaptation is characterized by the prioritization of external needs over internal ones, often leading to physical and psychological exhaustion through the suppression of self-feeling and over-compromising to maintain social cohesion or gain recognition (Abe et al., 2020). In contrast, empathic distress results from affective overload triggered by excessive emotional resonance with others’ suffering, leading to acute self-focused distress and subsequent withdrawal behaviors to prevent emotional exhaustion (O. Klimecki & Singer, 2011). Additionally, excessive adaptation is associated with long-term outcomes such as burnout and depressive symptoms due to persistent self-neglect, whereas empathic distress is more closely associated with immediate emotional dysregulation and reduced prosocial motivation.

The results of this study partially support Hypothesis 2, finding that excessive adaptation mediates the paths from empathic concern to its negative outcomes and prosocial behaviors. Excessive adaptation-prone individuals may become overly dependent on external feedback for emotional adjustments, neglecting the cultivation of their internal emotional regulation system, which may lead to depression (Ishizu & Ambo, 2008). When external feedback falls short of expectations, individuals may experience helplessness and anger, and this emotional instability can lead to an increase in reactive aggression. In terms of prosocial behaviors, Kuwayama (2003) points out that individuals who are over-adaptive are sensitive to the desires of others, and they try to behave in a near-perfect way to meet the expectations of others. Therefore, excessive adaptation also mediates the path from empathic concern to prosocial behaviors.

However, the relationship between excessive adaptation and positive mental health did not reach a significant level in the mediation model, and the null hypothesis was supported in the Bayesian regression analysis. Individuals with higher levels of excessive adaptation may focus extensively on meeting external demands, neglecting their personal needs and emotions. This excessive adaptation to the external environment may lead to a series of psychological problems, such as lower self-esteem (Fujimoto & Kira, 2014) and higher depression (Kazama, 2015). Therefore, excessive adaptation does not promote positive mental health but instead leads to depression, as it causes individuals to suppress their emotions and internal needs in the long term in order to meet external expectations.

The current findings contribute to the empathy–altruism hypothesis, by reflecting a significant positive correlation between empathic concern and prosocial behaviors. Altruistic behaviors are an indicator of mental health and social well-being (Liu et al., 2023), while empathic concern enables individuals to pay more attention to the feelings and needs of others, stimulating prosocial behaviors and thereby promoting altruism. In contrast, individuals with high levels of empathic concern do not exacerbate depression, which does not support the vulnerability model. By introducing excessive adaptation as a mediating variable, it reveals the internal mechanism of how empathic concern exerts its influence in different aspects, providing a more comprehensive framework for exploring and understanding the complexity of empathic concern.

This study has several implications for the medical field. Studies have argued that empathic concern is one of the key characteristics in establishing therapeutic relationships between nurses and patients (Forchuk et al., 1998; Gerace et al., 2018; McAllister et al., 2019), allowing clinicians to identify, understand, and empathize with their patients’ suffering (Guidi & Traversa, 2021), leading to better clinical outcomes (Reynolds & Scott, 1999). However, excessive empathic concern can lead to compassion fatigue, resulting in emotional exhaustion, helplessness, and loss of job satisfaction (Li et al., 2022; Xie et al., 2021). In addition, compassion fatigue can trigger anxiety and depression, reducing the quality of care (Salyers et al., 2015) and compromising their resilience (Nolte et al., 2017). In a word, effective strategies should be developed to prevent compassion fatigue in clinical settings, thereby maintaining the mental health of healthcare professionals.

The findings also contribute to education. Studies have indicated a negative correlation between excessive adaptation and school adjustment (Ishizu & Ambo, 2008; Mashiko, 2008). Adolescents with excessive adaptation often behave obediently and act in accordance with the wishes of their parents and teachers, making it difficult to identify problems in a timely manner. However, this seemingly positive state is often maintained at the expense of their internal needs, which may impair their school adjustment (Ishizu & Ambo, 2008; Mashiko, 2008) and social adaptation (W. Wang et al., 2021). Educators should be alert to this type of excessive adaptation and be aware of its potential dangers. Educators can use mental health education programmes and structured activities to guide and intervene with students to identify and express their inner needs. In addition, care should be taken to prevent excessive adaptation while fostering positive behaviors through empathic concern in related lessons. These interventions help students not only to alleviate the psychological pressures caused by excessive adaptation in the short term, but also to rebuild adolescents’ self-identity and need-expressing mechanisms, which ultimately promote the dynamic balance of their social adaptability.

This study has certain shortcomings. First, the findings are based on a cross-sectional design. Although the hypotheses are based on a solid theoretical and research foundation, there is no strong evidence to infer causal relationships. Future research should employ a longitudinal design to gather more substantial evidence. Second, the pathway from excessive adaptation to positive mental health did not reach a significant level; future studies might consider using a more diverse range of measures to assess mental health. Third, there is a sex imbalance in the sample, with a predominance of female participants. However, the inclusion of sex as a control variable in our models helps to mitigate potential biases. Nevertheless, the potential impact of gender on this model is worthy of further discussion. Additionally, the consistency of our findings with those from other studies that have more balanced samples further supports the robustness and generalizability of our results.

Last but not least, the present study did not examine other potential moderators, such as cultural background, developmental age, and socioeconomic status. The interaction between these factors may influence the manifestation of empathic concern in behavioral outcomes. Future research should systematically examine these contextual factors to test for generalizability across groups. For example, there is evidence that perspective-taking can foster an individual’s empathic concern for the suffering of others (Kang et al., 2025; Oswald, 1996). Therefore, it is important to explore how potential moderators like perspective-taking can buffer the effects of empathic concern on mental health maladjustment.

## Figures and Tables

**Table 1:** Pearson correlations, means, and standard deviations of all variables.

Variables	M	SD	1	2	3	4	5	6
1. Empathic concern	20.49	3.56	1					
2. Excessive adaption	69.58	13.51	0.122 ***	1				
3. Prosocial behaviors	97.77	17.17	0.331 ***	0.317 ***	1			
4. Reactive aggression	24.23	12.28	−0.372 ***	0.218 ***	−0.188 ***	1		
5. Depression	6.86	5.53	−0.248 ***	0.285 ***	−0.185 ***	0.617 ***	1	
6. Positive mental health	16.75	6.06	0.137 ***	−0.013	0.401 ***	−0.130 ***	−0.396 ***	1

Note. *** *p* < 0.001.

**Table 2:** Indirect effects for the mediating role of excessive adaptation.

Path	*b*	95% CI
empathic concern → excessive adaptation → prosocial behaviors	0.037	0.102, 0.257
empathic concern → excessive adaptation → positive mental health	−0.005	−0.021, 0.004
empathic concern → excessive adaptation → reactive aggression	0.035	0.067, 0.171
empathic concern → excessive adaptation → depression	0.042	0.038, 0.093

Note. Parameter *b* refers to the standardized path coefficient, and CI refers to the confidence intervals.

## Data Availability

The data sets used in this study are available from the corresponding author on reasonable request.

## References

[B1-behavsci-15-00463] Abe N., Abe K., Nakashima K. (2020). The role of perceived stress and fear of negative evaluation in the process from alexithymia to over-adaptation. Psychologia.

[B2-behavsci-15-00463] Allen J. J., Anderson C. A., Bushman B. J. (2018). The general aggression model. Current Opinion in Psychology.

[B3-behavsci-15-00463] Anderson C. A., Bushman B. J. (2002). Human aggression. Annual Review of Psychology.

[B4-behavsci-15-00463] Asai K. (2014). Effects of over-adaptation on subjective well-being in adolescence. The Japanese Journal of Psychology.

[B5-behavsci-15-00463] Astington J. W., Hughes C. (2013). Theory of mind: Self-reflection and social understanding. The Oxford handbook of developmental psychology, vol. 2: Self and other.

[B6-behavsci-15-00463] Atkins D., Uskul A. K., Cooper N. R. (2016). Culture shapes empathic responses to physical and social pain. Emotion.

[B7-behavsci-15-00463] Batson C. D. (2023). Empathic concern: What it is and why it’s important.

[B8-behavsci-15-00463] Batson C. D., Batson J. G., Slingsby J. K., Harrell K. L., Peekna H. M., Todd R. M. (1991). Empathic joy and the empathy-altruism hypothesis. Journal of Personality and Social Psychology.

[B9-behavsci-15-00463] Berking M., Whitley B. (2014). Affect regulation training: A practitioners’ manual.

[B10-behavsci-15-00463] Carlo G., Randall B. A. (2002). The development of a measure of prosocial behaviors for late adolescents. Journal of Youth and Adolescence.

[B11-behavsci-15-00463] Chierchia G., Singer T. (2017). The neuroscience of compassion and empathy and their link to prosocial motivation and behavior. Decision neuroscience.

[B12-behavsci-15-00463] Cicchetti D., Toth S. L. (2009). A developmental psychopathology perspective on adolescent depression. Handbook of depression in adolescents.

[B13-behavsci-15-00463] Coutinho J. F., Silva P. O., Decety J. (2014). Neurosciences, empathy, and healthy interpersonal relationships: Recent findings and implications for counseling psychology. Journal of Counseling Psychology.

[B14-behavsci-15-00463] Crocker J., Canevello A., Breines J. G., Flynn H. (2010). Interpersonal goals and change in anxiety and dysphoria in first-semester college students. Journal of Personality and Social Psychology.

[B15-behavsci-15-00463] Cuff B. M. P., Brown S. J., Taylor L., Howat D. J. (2016). Empathy: A review of the concept. Emotion Review.

[B16-behavsci-15-00463] Davis M. H. (1983a). Empathic concern and the muscular dystrophy telethon: Empathy as a multidimensional construct. Personality and Social Psychology Bulletin.

[B17-behavsci-15-00463] Davis M. H. (1983b). Measuring individual differences in empathy: Evidence for a multidimensional approach. Journal of Personality and Social Psychology.

[B18-behavsci-15-00463] Decety J., Cowell J. M. (2014). The complex relation between morality and empathy. Trends in Cognitive Sciences.

[B19-behavsci-15-00463] Decety J., Jackson P. L. (2004). The functional architecture of human empathy. Behavioral and Cognitive Neuroscience Reviews.

[B20-behavsci-15-00463] Decety J., Lamm C., Decety J., Ickes W. (2009). Empathy versus personal distress: Recent evidence from social neuroscience. The social neuroscience of empathy.

[B21-behavsci-15-00463] Decety J., Svetlova M. (2012). Putting together phylogenetic and ontogenetic perspectives on empathy. Developmental Cognitive Neuroscience.

[B22-behavsci-15-00463] De Waal F. B. M., Preston S. D. (2017). Mammalian empathy: Behavioural manifestations and neural basis. Nature Reviews Neuroscience.

[B23-behavsci-15-00463] Ding D., Tan X., Sun L., Zeng X., Yan Z. (2024). Does empathy lead to depression? A three-wave random intercept cross-lagged panel analysis in emerging adults. Current Psychology.

[B24-behavsci-15-00463] Dodge K. A., Coie J. D. (1987). Social-information-processing factors in reactive and proactive aggression in children’s peer groups. Journal of Personality and Social Psychology.

[B25-behavsci-15-00463] Eisenberg N., Spinrad T. L., Eggum N. D. (2010). Emotion-related self-regulation and its relation to children’s maladjustment. Annual Review of Clinical Psychology.

[B26-behavsci-15-00463] Euler F., Steinlin C., Stadler C. (2017). Distinct profiles of reactive and proactive aggression in adolescents: Associations with cognitive and affective empathy. Child and Adolescent Psychiatry and Mental Health.

[B27-behavsci-15-00463] Figley C. R. (1995). Compassion fatigue: Toward a new understanding of the costs of caring. Secondary traumatic stress: Self-care issues for clinicians, researchers, and educators.

[B28-behavsci-15-00463] Forchuk C., Westwell J., Martin M.-L., Azzapardi W. B., Kosterewa-Tolman D., Hux M. (1998). Factors influencing movement of chronic psychiatric patients from the orientation to the working phase of the nurse-client relationship on an inpatient unit. Perspectives in Psychiatric Care.

[B29-behavsci-15-00463] Fu W., Wang C., Chai H., Xue R. (2022). Examining the relationship of empathy, social support, and prosocial behavior of adolescents in China: A structural equation modeling approach. Humanities and Social Sciences Communications.

[B30-behavsci-15-00463] Fujimoto S., Kira Y. (2014). The study of over-adaptation and self-esteem in adolescence. Faculty of Human-Environment Studies, Kyushu University.

[B31-behavsci-15-00463] Gambin M., Sharp C. (2018). The relations between empathy, guilt, shame and depression in inpatient adolescents. Journal of Affective Disorders.

[B32-behavsci-15-00463] Gerace A., Oster C., O’Kane D., Hayman C. L., Muir-Cochrane E. (2018). Empathic processes during nurse–consumer conflict situations in psychiatric inpatient units: A qualitative study. International Journal of Mental Health Nursing.

[B33-behavsci-15-00463] Gill K. L., Calkins S. D. (2003). Do aggressive/destructive toddlers lack concern for others? Behavioral and physiological indicators of empathic responding in 2-year-old children. Development and Psychopathology.

[B34-behavsci-15-00463] Gleichgerrcht E., Decety J. (2013). Empathy in clinical practice: How individual dispositions, gender, and experience moderate empathic concern, burnout, and emotional distress in physicians. PLoS ONE.

[B35-behavsci-15-00463] Gong X., Xie X., Xu R., Luo Y. (2010). Psychometric properties of the Chinese versions of DASS-21 in Chinese college students. Chinese Journal of Clinical Psychology.

[B36-behavsci-15-00463] Greene J. D., Nystrom L. E., Engell A. D., Darley J. M., Cohen J. D. (2004). The neural bases of cognitive conflict and control in moral judgment. Neuron.

[B37-behavsci-15-00463] Guidi C., Traversa C. (2021). Empathy in patient care: From ‘clinical empathy’ to ‘empathic concern’. Medicine, Health Care and Philosophy.

[B38-behavsci-15-00463] Guo X., Zheng H., Ruan D., Hu D., Wang Y., Wang Y., Raymond C. K. C. (2023). Associations between empathy and negative affect: Effect of emotion regulation. Acta Psychologica Sinica.

[B39-behavsci-15-00463] He Y., Yang L., Qian C., Li T., Su Z., Zhang Q., Hou X. (2023). Conversational agent interventions for mental health problems: Systematic review and meta-analysis of randomized controlled trials. Journal of Medical Internet Research.

[B40-behavsci-15-00463] Hong S., Wang Z. (2024). Understanding the impact of expertise on compassion fatigue in counseling via core self-evaluation and resilience. Scientific Reports.

[B41-behavsci-15-00463] Hoyle R. H. (2012). Handbook of structural equation modeling.

[B42-behavsci-15-00463] Huesmann L. R. (2018). An integrative theoretical understanding of aggression: A brief exposition. Current Opinion in Psychology.

[B43-behavsci-15-00463] Ishizu K., Ambo H. (2008). Tendency toward over-adaptation: School adjustment and stress responses. The Japanese Journal of Educational Psychology.

[B44-behavsci-15-00463] Jackson D. C., Malmstadt J. R., Larson C. L., Davidson R. J. (2000). Suppression and enhancement of emotional responses to unpleasant pictures. Psychophysiology.

[B45-behavsci-15-00463] Jarosz A. F., Wiley J. (2014). What are the odds? A practical guide to computing and reporting Bayes factors. The Journal of Problem Solving.

[B46-behavsci-15-00463] Jeffreys H. (1961). Theory of probability.

[B47-behavsci-15-00463] Kang Y., Mesquiti S., Baik E. S., Falk E. B. (2025). Empathy and helping: The role of affect in response to others’ suffering. Scientific Reports.

[B48-behavsci-15-00463] Kazama J. (2015). The relationship between over-adaptation and depression in college students. The Japanese Journal of Adolescent Psychology.

[B49-behavsci-15-00463] Kenny D. A., Korchmaros J. D., Bolger N. (2003). Lower level mediation in multilevel models. Psychological Methods.

[B52-behavsci-15-00463] Klimecki O., Singer T., Oakley B., Knafo A., Madhavan G., Wilson D. S. (2011). Empathic distress fatigue rather than compassion fatigue?. tegrating findings from empathy research in psychology and social neuroscience.

[B50-behavsci-15-00463] Klimecki O. M., Leiberg S., Lamm C., Singer T. (2013). Functional neural plasticity and associated changes in positive affect after compassion training. Cerebral Cortex.

[B51-behavsci-15-00463] Klimecki O. M., Leiberg S., Ricard M., Singer T. (2014). Differential pattern of functional brain plasticity after compassion and empathy training. Social Cognitive and Affective Neuroscience.

[B53-behavsci-15-00463] Kosaka Y. (2008). Inferiority feeling and self-oriented perfectionism in adolescence. The Japanese Journal of Personality.

[B54-behavsci-15-00463] Kou Y., Wang L. (2003). The review of researches about prosocial behaviors and intervent training in children. Psychological Development and Education.

[B55-behavsci-15-00463] Kuwayama K. (2003). A study on over-adaptation by means of emotional expressions in the frustrating situations. Kyoto University Research Studies in Education.

[B56-behavsci-15-00463] Lebowitz M. S., Dovidio J. F. (2015). Implications of emotion regulation strategies for empathic concern, social attitudes, and helping behavior. Emotion.

[B57-behavsci-15-00463] Leiberg S., Klimecki O., Singer T. (2011). Short-term compassion training increases prosocial behavior in a newly developed prosocial game. PLoS ONE.

[B58-behavsci-15-00463] Levy J., Goldstein A., Feldman R. (2019). The neural development of empathy is sensitive to caregiving and early trauma. Nature Communications.

[B59-behavsci-15-00463] Li J., Wang Q., Guan C., Luo L., Hu X. (2022). Compassion fatigue and compassion satisfaction among Chinese palliative care nurses: A province-wide cross-sectional survey. Journal of Nursing Management.

[B60-behavsci-15-00463] Liu X., Zhang Y., Chen Z., Xiang G., Miao H., Guo C. (2023). Effect of socioeconomic status on altruistic behavior in Chinese middle school students: Mediating role of empathy. International Journal of Environmental Research and Public Health.

[B61-behavsci-15-00463] Lovett B., Sheffield R. (2007). Affective empathy deficits in aggressive children and adolescents: A critical review. Clinical Psychology Review.

[B62-behavsci-15-00463] Lovibond P. F., Lovibond S. H. (1995). The structure of negative emotional states: Comparison of the Depression Anxiety Stress Scales (DASS) with the beck depression and anxiety inventories. Behaviour Research and Therapy.

[B63-behavsci-15-00463] Lukat J., Margraf J., Lutz R., Van Der Veld W. M., Becker E. S. (2016). Psychometric properties of the Positive Mental Health Scale (PMH-scale). BMC Psychology.

[B64-behavsci-15-00463] MacKinnon D. P. (2012). Introduction to statistical mediation analysis.

[B65-behavsci-15-00463] Markus H. R., Kitayama S. (1991). Culture and the self: Implications for cognition, emotion, and motivation. Psychological Review.

[B66-behavsci-15-00463] Mashiko H. (2008). The relationship between the tendency of over-adaptation and personality trait, fears of abandonment, and approval motivation in adolescence. Japanese Journal of Counseling Science.

[B67-behavsci-15-00463] Mashiko H. (2009). The relationship between over-adaptation and depression, obsessive-compulsive symptoms, anthropophobic tendency and school nonattendance: Based on questionnaire data from two Japanese high schools. Journal of School Mental Health.

[B68-behavsci-15-00463] McAllister S., Robert G., Tsianakas V., McCrae N. (2019). Conceptualising nurse-patient therapeutic engagement on acute mental health wards: An integrative review. International Journal of Nursing Studies.

[B69-behavsci-15-00463] McGrath L. M., Weill S., Robinson E. B., Macrae R., Smoller J. W. (2012). Bringing a developmental perspective to anxiety genetics. Development and Psychopathology.

[B70-behavsci-15-00463] Moreno I. R., Comes-Fayos J., Bressanutti S., Blasco-Ros C., Martínez M., Lila M., Romero-Martínez Á., Moya-Albiol L. (2024). Unraveling the layers of empathy: Self-compassion as a partial mediator in male and female offenders’ personal distress and aggression. Journal of Aggression, Maltreatment & Trauma.

[B71-behavsci-15-00463] Nolte A. G., Downing C., Temane A., Hastings-Tolsma M. (2017). Compassion fatigue in nurses: A metasynthesis. Journal of Clinical Nursing.

[B72-behavsci-15-00463] Oswald P. A. (1996). The effects of cognitive and affective perspective taking on empathic concern and altruistic helping. The Journal of Social Psychology.

[B73-behavsci-15-00463] Patil I., Silani G. (2014). Reduced empathic concern leads to utilitarian moral judgments in trait alexithymia. Frontiers in Psychology.

[B74-behavsci-15-00463] Persson B. N., Kajonius P. J. (2016). Empathy and universal values explicated by the empathy-altruism hypothesis. The Journal of Social Psychology.

[B75-behavsci-15-00463] Pluta A., Mazurek J., Wojciechowski J., Wolak T., Soral W., Bilewicz M. (2023). Exposure to hate speech deteriorates neurocognitive mechanisms of the ability to understand others’ pain. Scientific Reports.

[B76-behavsci-15-00463] Podsakoff P. M., MacKenzie S. B., Lee J.-Y., Podsakoff N. P. (2003). Common method biases in behavioral research: A critical review of the literature and recommended remedies. Journal of Applied Psychology.

[B77-behavsci-15-00463] Qiu X., Gao M., Zhu H., Li W., Jiang R. (2024). Theory of mind, empathy, and prosocial behavior in children and adolescent: A meta-analysis. Current Psychology.

[B78-behavsci-15-00463] Raine A., Dodge K., Loeber R., Gatzke-Kopp L., Lynam D., Reynolds C., Stouthamer-Loeber M., Liu J. (2006). The reactive–proactive aggression questionnaire: Differential correlates of reactive and proactive aggression in adolescent boys. Aggressive Behavior.

[B79-behavsci-15-00463] Ren Y. J. (2022). Future tasks of study on over-adaptation in Japanese and Chinese adolescence: A reply KAZAMA’s and WAKAMATSU’s comments. Japanese Journal of Adolescent Psychology.

[B80-behavsci-15-00463] Reynolds W. J., Scott B. (1999). Empathy: A crucial component of the helping relationship. Journal of Psychiatric and Mental Health Nursing.

[B81-behavsci-15-00463] Ritchie M. B., Neufeld R. W. J., Yoon M., Li A., Mitchell D. G. V. (2022). Predicting youth aggression with empathy and callous unemotional traits: A Meta-analytic review. Clinical Psychology Review.

[B82-behavsci-15-00463] Rogers P. (2024). Best practices for your confirmatory factor analysis: A JASP and lavaan tutorial. Behavior Research Methods.

[B83-behavsci-15-00463] Salyers M. P., Fukui S., Rollins A. L., Firmin R., Gearhart T., Noll J. P., Williams S., Davis C. J. (2015). Burnout and self-reported quality of care in community mental health. Administration and Policy in Mental Health and Mental Health Services Research.

[B84-behavsci-15-00463] Schipper M., Petermann F. (2013). Relating empathy and emotion regulation: Do deficits in empathy trigger emotion dysregulation?. Social Neuroscience.

[B85-behavsci-15-00463] Shamay-Tsoory S. G. (2011). The neural bases for empathy. The Neuroscientist.

[B86-behavsci-15-00463] Song P., Zhang Z., Wang B., David N., Zhao H., Wang Q., Xiao Y., Yang B. (2018). The influence of trait empathy on reactive aggression: An ERP study. International Journal of Psychophysiology.

[B87-behavsci-15-00463] Stefanello E. (2022). Your pain is not mine: A critique of clinical empathy. Bioethics.

[B88-behavsci-15-00463] Strayer J., Roberts W. (2004). Empathy and observed anger and aggression in five-year-olds. Social Development.

[B89-behavsci-15-00463] Tampke E. C., Fite P. J., Cooley J. L. (2020). Bidirectional associations between affective empathy and proactive and reactive aggression. Aggressive Behavior.

[B90-behavsci-15-00463] Tone E. B., Tully E. C. (2014). Empathy as a “risky strength”: A multilevel examination of empathy and risk for internalizing disorders. Development and Psychopathology.

[B91-behavsci-15-00463] Tsuda Y. (2021). When a modest person becomes happy. The Proceedings of the Annual Convention of the Japanese Psychological Association.

[B92-behavsci-15-00463] Twenge J. M., Baumeister R. F., DeWall C. N., Ciarocco N. J., Bartels J. M. (2007). Social exclusion decreases prosocial behavior. Journal of Personality and Social Psychology.

[B93-behavsci-15-00463] Wang W., Wen L., Wang Y. (2021). The development characteristics, influencing factors, and promotion of children’s social adjustment. Studies in Early Childhood Education.

[B94-behavsci-15-00463] Wang X. (2021). A comparison of chinese and japanese junior school students’over-adaptation. Journal of Ningbo University (Educational Science Edition).

[B95-behavsci-15-00463] Wen Z., Ye B. (2014). Analyses of mediating effects: The development of methods and models. Advances in Psychological Science.

[B96-behavsci-15-00463] Wetzels R., Matzke D., Lee M. D., Rouder J. N., Iverson G. J., Wagenmakers E.-J. (2011). Statistical evidence in experimental psychology: An empirical comparison using 855 t tests. Perspectives on Psychological Science.

[B97-behavsci-15-00463] Wölfer R., Cortina K. S., Baumert J. (2012). Embeddedness and empathy: How the social network shapes adolescents’ social understanding. Journal of Adolescence.

[B98-behavsci-15-00463] Xie W., Chen L., Feng F., Okoli C. T. C., Tang P., Zeng L., Jin M., Zhang Y., Wang J. (2021). The prevalence of compassion satisfaction and compassion fatigue among nurses: A systematic review and meta-analysis. International Journal of Nursing Studies.

[B99-behavsci-15-00463] Zaki J. (2020). Integrating empathy and interpersonal emotion regulation. Annual Review of Psychology.

[B100-behavsci-15-00463] Zhang F. F., Dong Y., Wang K. (2010). Reliability and validity of the Chinese version of the Interpersonal Reactivity Index-C. Chinese Journal of Clinical Psychology.

[B101-behavsci-15-00463] Zhang M., Wang S., Wang Z., Peng X., Fei W., Geng Y., Zhang T. (2021). Associations of affective and cognitive empathy with depressive symptoms among a sample of Chinese college freshmen. Journal of Affective Disorders.

[B102-behavsci-15-00463] Zhang W. L., Jia S. W., Chen G. H., Zhang W. X. (2014). Reliability and validity of reactive—Proactive aggression questionnaire in college students. Chinese Journal of Clinical Psychology.

[B103-behavsci-15-00463] Zhang Y.-Y., Han W.-L., Qin W., Yin H.-X., Zhang C.-F., Kong C., Wang Y.-L. (2018). Extent of compassion satisfaction, compassion fatigue and burnout in nursing: A meta-analysis. Journal of Nursing Management.

[B104-behavsci-15-00463] Zheng S., Tanaka R., Ishii K. (2024). Empathic concern promotes social support-seeking: A cross-cultural study. Emotion.

